# Palliative care progress in Benin: a situation analysis using the WHO development indicators

**DOI:** 10.1186/s12904-024-01473-9

**Published:** 2024-06-05

**Authors:** Kouessi Anthelme Agbodande, Freddy Gnangnon, Mickael Assogba, Josué Avakoudjo, Angèle Azon Kouanou, Lisette Odoulamy, Jean Daho, Djimon Marcel Zannou, Sourakatou Salifou, Ali Imorou Bah Chabi, Raoul Saizonou, Issimouha Dille Mahamadou, Fernanda Bastos, Eduardo Garralda, Carlos Centeno, Vilma Adriana Tripodoro

**Affiliations:** 1 Programme National des soins Palliatifs (PNSP/MS), Cotonou, Bénin; 2 Service de médecine interne -Centre National Hospitalo-Universitaire CNHU-HKM, Cotonou, Benin; 3 Médecin Point focal Cancer FSS/UAC/PNLMNT/MS, Cotonou, Benin; 4 SoBECAN: Société Béninoise de lutte contre le Cancer, Cotonou, Benin; 5 Unité de Soins Palliatifs -Centre National Hospitalo-Universitaire CNHU-HKM, Cotonou, Benin; 6 Faculté des Sciences de la Santé, Cotonou, Benin; 7 Association Béninoise de soins palliatifs (ABSP), Cotonou, Bénin; 8 Institut National Médico-Sanitaire (INMeS), Cotonou, Benin; 9 Directeur Départemental de la santé du Couffo, Aplahoué, Benin; 10 Conseil National de la Médecine Hospitalière (CNMH), Cotonou, Benin; 11 Directeur National de la Santé Publique (DNSP), Cotonou, Benin; 12 Secrétaire Général du Ministère SGM/MS, Cotonou, Benin; 13 National Professional Officier/NCD; OMS, Cotonou, Benin; 14 Médecin Technical officer Cancer for West and Central, Africa OMS/AFRO Unité MNT/AFRO Cluster (UCN), Dakar, Sénégal; 15 ATLANTES Global Observatory of Palliative Care, Institute Culture and Society, University of Navarra, Navarra, Spain

**Keywords:** Indicators, Palliative care, Assessment, Global health, Benin, Africa

## Abstract

**Context:**

Palliative care (PC) in most African countries remains under-assessed. Benin has piloted the implementation of a set of indicators proposed by the WHO to measure PC development.

**Objectives:**

To examine the current status of PC in Benin.

**Methods:**

A workshop with stakeholders was organized to assess the WHO indicators in the Beninese context. Indicators were rated based on relevance and feasibility, data sources were agreed upon, and a survey was adapted. Data were collected between March and May 2023.

**Results:**

There is emerging community involvement in PC through the presence of patients’ rights promoters, as well as a political commitment expressed in the National PC strategy, the inclusion of PC services in the list of basic health services, and an assigned national authority –within the Ministry of Health–responsible for PC. Although no PC-oriented research has been documented, the celebration of the National PC Conference represents the first step to ground PC delivery in evidence. The reported annual consumption of opioids is 0.18 (ME) milligrams per capita, 34% of healthcare establishments have essential medicines for pain and PC, and 16.5% of patients with palliative needs have access to oral morphine. To date, no medical or paramedical schools offer PC training, and there is no official specialization in palliative medicine for doctors. PC is provided by 11 specialist teams (0.08/100,000 inhabitants), none of which provides pediatric care.

**Conclusion:**

Despite growing political, professional, and community commitments to palliative care, there are challenges in education, research, essential medicines, and access to PC services.

**Supplementary Information:**

The online version contains supplementary material available at 10.1186/s12904-024-01473-9.

## Key message

This article describes the first macro-level assessment of the status of palliative care development in Benin. A set of indicators adopted by the World Health Organization has enabled an understanding of the gaps in education, essential medicines, and specialized services for an adequate provision of palliative care.

**Supplementary Information:**

The online version contains supplementary material available at 10.1186/s12904-024-01473-9.

## Introduction

Palliative care has been recognized as a human right and should be provided as part of integrated, people-centered health services, paying special attention to the specific needs and preferences of individuals [1]. However, according to the World Health Organization, 40 million people need palliative care each year, and 78% of them live in low- and middle-income countries. Globally, only about 14% of people in need of palliative care currently receive it [2].

To estimate the need for palliative care, a Lancet commission report published in 2018 identified 20 health conditions that most commonly result either in death or in suffering that is severe enough to require palliative care intervention for people of any age across the globe [3]. In sub-Saharan Africa, the burden of HIV, tuberculosis, cancer, and other NCDs is particularly great [4]. Moreover, there is growing concern that people’s lifestyles, food preferences, and sedentary working habits on the continent are changing. Unhealthy lifestyles could lead to an increase in the incidence of life-threatening chronic diseases in Africa, thereby increasing the need for palliative care [5].

In Benin, non-communicable diseases constitute the most common cause of death, representing 53.36% of all deaths. The prevalence of cardiovascular diseases is estimated at 25.90%, and the most frequent cancers are breast cancer (32,5%) and cervical cancer (16.8%) [6–8]. Recent data from 2020 estimate that the number of cancer cases in Benin is 11,548, with a total of 4,662 cancer-related deaths [6]. Regarding some of the most important communicable diseases that would benefit from palliative care, the prevalence of HIV and tuberculosis is estimated at 1.2% and 12%, respectively. According to the Ministry of Health of Benin, 62,531 people are estimated to require PC [8]. In 2022, the Lancet Oncology Commission recommended supportive and palliative care to update the national cancer control plans in sub-Saharan Africa [9]. 

**Fig. 1:**
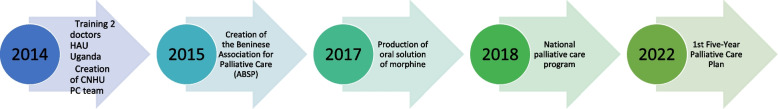
Main milestones in the historical development of palliative care in Benin

Coinciding with the 2014 World Health Assembly resolution WHA67.19, calling on Member States to develop palliative care policies [8], the history of palliative care in Benin started with the training in Uganda of two medical doctors from the Internal Medicine department at National Center University Hospital Hubert K. Maga (CNHU HKM) (Fig. 1). They established the first two teams (at the CNHU-HKM in Cotonou and at the Comè Zone Hospital) and the Benin Palliative Care Association (ABSP) with the support of committed volunteers. Through the leadership of the pioneers, in 2017, a unit for the production of oral morphine solution from morphine powder was established. In September 2018, in the application of the 2014 resolution WHA 67.19, the government of Benin took ownership of the initiative, which fits in with ongoing health reforms. Thus, on September 12, 2018, the Council of Ministers of Benin decided to create the National Palliative Care Program (PNSP). This Five-Year Palliative Care Plan was developed in Benin in 2022, aiming to make the country a palliative care model that can be implemented not only in Benin but also adapted to sub-Saharan African realities [8].

Despite these developments and the clear commitment of palliative care pioneers, an evaluation of the current situation that builds on previous evaluations [10, 11] would allow a better understanding of the degree of preparedness of Benin to match the population´s palliative care needs. Using the report ‘Assessing the development of palliative care worldwide: a set of actionable indicators’ [12] published in 2021, that provides a globally applicable set of indicators, the objective of this study is to describe the evaluation process and current situation of the development of palliative care in Benin.

## Methods

The evaluation process was led by the WHO Department of Integrated Health Services Geneva, AFRO Regional Office, and country office of Benin. These commissioned the technical evaluation to a WHO collaborating center (The ATLANTES Global Observatory of Palliative Care) and in-country stakeholders. It serves as a pilot for monitoring palliative care based on the WHO report on indicators [12].

The set of indicators contained in the WHO report served as a starting point from which a six-step process was contemplated: 1st ) meeting with Benin´s key stakeholders to explain, validate, and adapt the indicators to the country’s needs, 2nd ) alignment with the country’s health information system, 3rd ) implementation of data collection/generation, 4th ) use of the data through a national report identifying areas for quality improvement and recommendations to strengthen palliative care, 5th ) end-of-project meeting with stakeholders to review the results, and 6th ) action plan. In this article, we summarize the first three steps of the results of the evaluation of palliative care development in Benin.

### Indicators

A 2-day workshop with academic support institutions, national professional associations, program coordinators from the Ministry of Health, including the Department of Health Information, representatives from WHO country offices, and NGOs, was held in Benin in February 2023 (Supplementary Table 1).

**Fig. 2:**
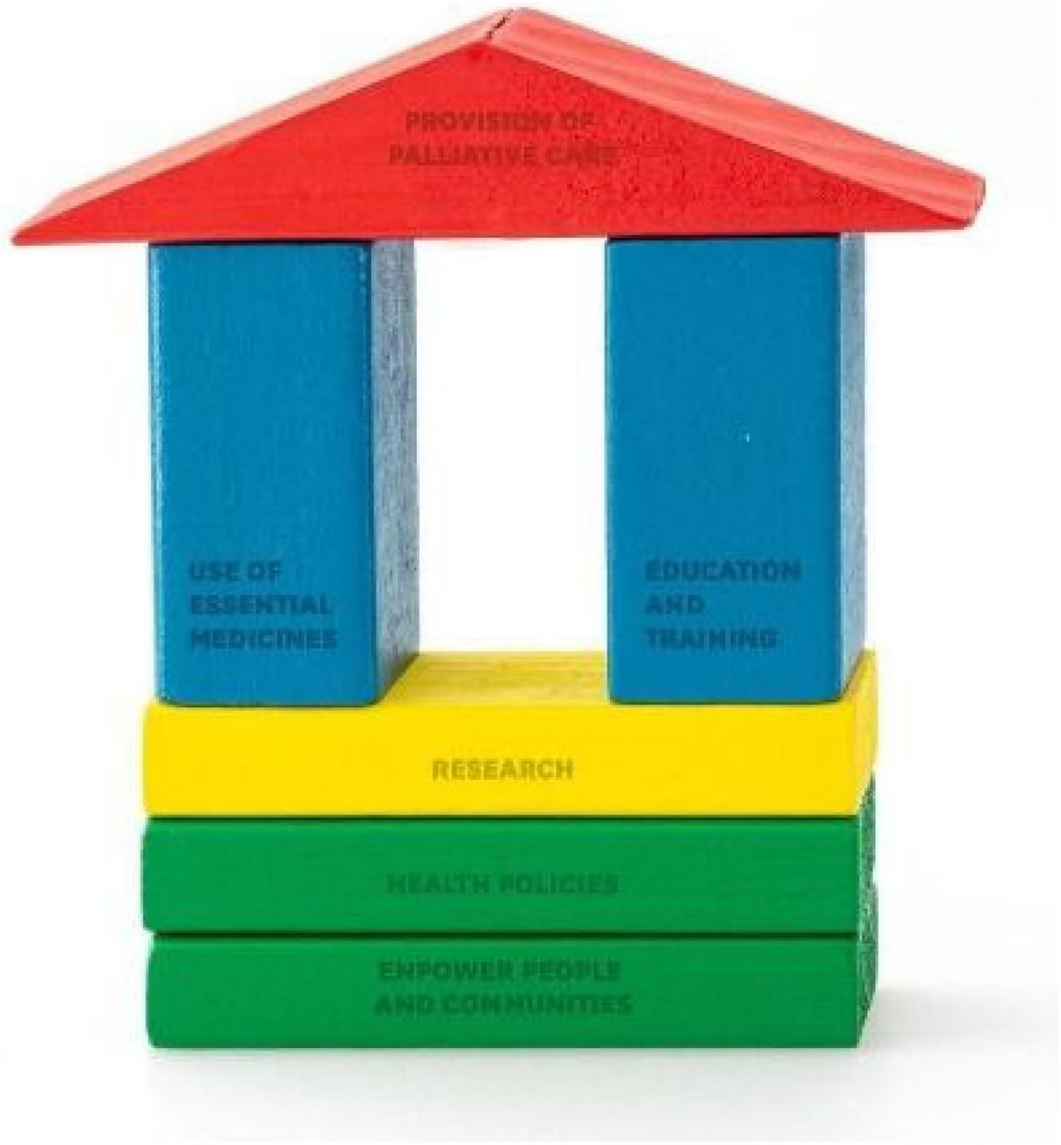
WHO conceptual model

During the first day of the workshop, ATLANTES explained the new WHO conceptual framework for the development of palliative care (Fig. 2) and the concepts behind all 14 indicators contained in the WHO report (Supplementary Table 2), one by one, with 30 participants.

This helped to understand, clear up ambiguities, and adapt the indicators culturally and linguistically, as well as to gain an overall validation of all six dimensions of the new WHO conceptual model for palliative care development.

On the second day, in order to understand both the relevance and feasibility of the country, a shortened list of 13 stakeholders representative of the Ministry of Health, the World Health Organization, NGOs, the National NCDs Program, main clinics, and the National PC Association voted on the relevance (R) and feasibility (F) of the indicators. They were scored on a Likert scale of 1 to 5 (1 = least, 5 = most) using *SurveyMonkey*. Statistical measures such as the median, content validity index (capacity to evaluate the “development” construct) and disagreement index (dispersion measure to understand homogeneity of scores) were calculated. The content validity index was calculated following Lawshe et al. using the following formula: ((n voters with highest scores: 4 and 5) – (n total voters/2)) / (n total voters / 2). The score ranges from 0 to 1 and should be interpreted as adequate if it is above 0.80. The Disagreement index (symmetry-adjusted inter-percentile rank) was calculated following Fitch et al. as the ratio between the inter-percentile rank (IPR) and the symmetry-adjusted inter-percentile rank (SAIPR). The IRP is calculated as the difference between the 70th percentile and the 30th percentile and the IPRAS using the following formula: IPRAS = 2.35 + 1.5*IA, where IA is the asymmetry index, representing the distance between the centre point of the RPI and the value 5 (centre point of the rating scale from 1 to 9) The score ranges from 0 to 1 and should be interpreted in the following way: disagreement increases as the value moves away from 0.

Although different scores were given by participants in terms of relevance (R) and feasibility (F), scores were high for all statistics calculated: median (*R* = 5, F = 4), content validity index (*R* = 0.85, F = 0.23), and the disagreement index (*R* = 0.09, F = 0.24) (Supplementary Table 1).

### Data sources

Available sources of information were discussed during the country meeting to align with existing country information systems. The different authorities and representatives discussed the best possible and precise data sources for every indicator to reach an agreement (Supplementary Table 3).

### Data collection

A survey exploring all indicators was prepared and adapted in French, considering one new indicator (indicator 7.1 of Supplementary Table 2) and cultural/language issues throughout the questions. Questions were edited to the best understanding of the Beninese context and had a scale to respond according to the level of development of that particular question: early, intermediate, established, or advanced (see full *survey*). These levels provide an in-depth description of their requirements and is the result of a consensus of the PHC department at WHO Geneva and the ATLANTES Global Observatory of Palliative Care, based on previous progress categorisations (Clark et al., 2020). Data were collected by the consultant (FG) with the help of the Coordinator of the National PC Program (AA) following established data sources (Supplementary Table 2).

Data were collected using a questionnaire designed for the needs of the study (based on the indicators from an international Delphi consensus process) [12], and were therefore used for the first time. The questionnaire results are shown in Supplementary Table 4. For the majority of the questions, stakeholders (as detailed in Supplementary Table 3) were contacted by e-mail, telephone, and by a presential visit to the head office of the institution when necessary (Coordinator of the National PC Programme and focal point of the NCDs strategy for indicator 1, and Deans for the Faculty of Health Sciences and National Nursing Institute for indicators 11, and 12). In particular, for the availability of palliative care drugs, a questionnaire (Word format) was sent electronically to all head doctors at the health facilities. They were contacted twice, a week apart.

All the data were retrieved in an Excel file using in all cases strict security measures to guarantee confidentiality. The completed forms were stored securely on a password-protected computer at the *Laboratoire d’épidémiologie des maladies chroniques et neurologiques* (LEMACEN) in the Faculty of Health Sciences. Access was restricted to the consultant.

### Data analysis

Data from the excel sheet were analysed using simple descriptive statistics or narratives depending on the indicator. A tentative level of development was assigned based on the value of the indicator (categories were defined in the full *survey).* For indicators with several attributes, the median was calculated to assign an overall level for the indicator. Indicators with numerical responses and levels of development were classified as follows: *indicator 7* (advanced: >2 articles/100,000 inhabitants; established: 1–2; intermediate: 0,31 − 1; and Early: 0–0,30), *indicator 8* (Advanced ≥ 100 mg/capita/year; established: 100 − 30; intermediate: 30 − 10; and early: < 10) for indicators *9, 10, and 11* (advanced: 70–100%; established 30–70%; intermediate: 10–30%; early: 0–10%).

The assignment of this level was justified and discussed with the technical team (ATLANTES) in weekly meetings between February and April 2023. In addition to the level of development, a justification using either quantitative or qualitative data was required to assess the final data. As a result, the current situation of palliative care development can be seen in the upcoming paragraphs, divided by the six elements of the conceptual PC Framework.

## Results

The main results are presented as a narrative by the WHO dimensions of PC development following the house model. The findings are summarized in Table 1.

**Table 1.**
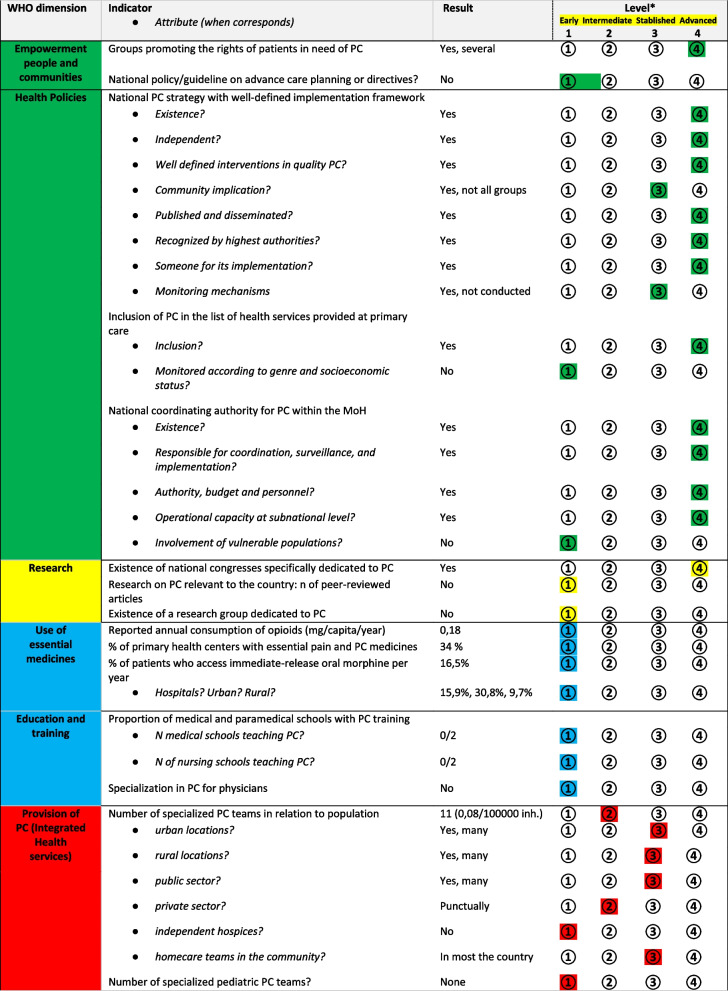
Main data reported on indicators & level of development in Benin

*Levels of development: 1 Early, 2 Intermediate, 3 Stablished, 4 Advanced

### Empowerment of people and communities

In Benin, there are several associations involved at both national and local levels in the defense of patients’ rights to palliative care such as cancer patient’s associations like *SOS Cancer Bénin* or the «*Fondation SPH contre le cancer*. There is also a specialized professional association, the “*Association Béninoise de Soins Palliatifs* (ABSP),” created in 2015, following the model of Hospice Africa Uganda. The ABSP aims to promote PC by integrating clinical, psychosocial, and scientific aspects. They coordinated information, education, and training activities for health structures to develop PC practices.

Regarding patient care planning, there are no specific policies for PC, such as advance directives, substitute decision makers, living wills, or advance care planning. Guidelines are being developed by the National Program for Palliative Care (PNSP), but have not yet been validated by the Ministry of Health. However, there is a law on the protection of the health of persons in the Republic of Benin that ensures the right of patients to information, to accept or refuse treatments and medical interventions, informed consent, and patient´s autonomy in any decision [13].

### Policies

Benin has an independent national strategic plan for palliative care, including an implementation plan and budget: The Five-Year Palliative Care Plan in Benin (PQSP 2022–2026). This plan is recent and has not yet been actively evaluated. It constitutes a reference framework for activities to be carried out to improve the quality of life of people with potentially incurable diseases, reflecting the Beninese government’s desire to fulfil its commitments to implement the Sustainable Development Goals (SDGs).

Article 36 of the National Health law (2020-37 of February 3, 2021, on the protection of people’s health in the Republic of Benin) [13] stipulates that the state promotes access to palliative care, meaning that PC is included in the list of basic health services to be provided to the population. Additionally, there is a module devoted to palliative care in the training guide for community workers at the national level. This guide is written by the National Council for the Fight against HIV/AIDS, Tuberculosis, Malaria, Hepatitis, Sexually Transmitted Infections, and Epidemics (CNLS-TP), which is directly attached to the Presidency of the Republic of Benin [14].

The National Palliative Care Program is within the structure of the Ministry of Health and works to prevent and alleviate the suffering of adult and child patients facing problems related to life-threatening diseases. The National Palliative Care Program is headed by a coordinator appointed by the Minister of Health.

### Research

The first Beninese National Palliative Care Congress was held from October 6 to 8, 2022 under the theme “Palliative care, universal health coverage and development.” It was coupled with the celebration of the Second World Palliative Care Day in Benin with the theme “Healing Hearts and Communities” and is planned to be conducted every year. The 2022 Congress was jointly organized by the National Palliative Care Program (PNSP) and the Beninese Association for Palliative Care (ABSP) under the sponsorship of the Beninese Minister of Health. Although no publications specific to palliative care were found in indexed databases, a new publication published as an abstract in the abstract book is about to be published [15, 16], and there is some clinical literature concerning CNMU HKM [17] and a project to create a PC-related research group led by PNSP.

### Use of medicines

According to the INCB, the reported annual consumption of opioids, excluding methadone, in oral morphine equivalents per person in Benin in 2020 was 0.18 mg/capita/year. Of this amount, morphine represents 0.14 mg, fentanyl 0.03 and pethidine 0.01, according to data from the *Walther Global Palliative Care Center (Indiana University)* website. There was a peak in morphine consumption in 2015.

Coordinators of health areas reported that 34% of the healthcare centers across the country had pain and PC medicines available. Non-opioid analgesics were available at 98.3% of health centers and non-steroidal anti-inflammatory drugs were available at 99% (512/521 and 514/521, respectively), while opioids were available at 6.3% of healthcare centers (33/521). In particular, oral morphine is available at those few centers where PC is provided, and 16.5% of patients followed at the hospital have access to oral morphine. Of the 44 hospitals, seven (15.9%) reported the availability of oral morphine, with a greater availability in urban hospitals (4/13:30.8%) than in rural ones (3/31,9.7%).

### Education

To date, neither of the two university institutions have a compulsory or optional palliative care module in their basic training. However, the Faculty of Medicine in Parakou is planning a module for palliative care from 2024. In addition, at the National Medical and Health Institute (INMeS/UAC), a training school for nurses and midwives, a master’s degree in palliative care was created in August 2021, but is not primarily intended for doctors. There is no accredited specialization course in palliative medicine for physicians.

### Provision of services

**Fig. 3:**
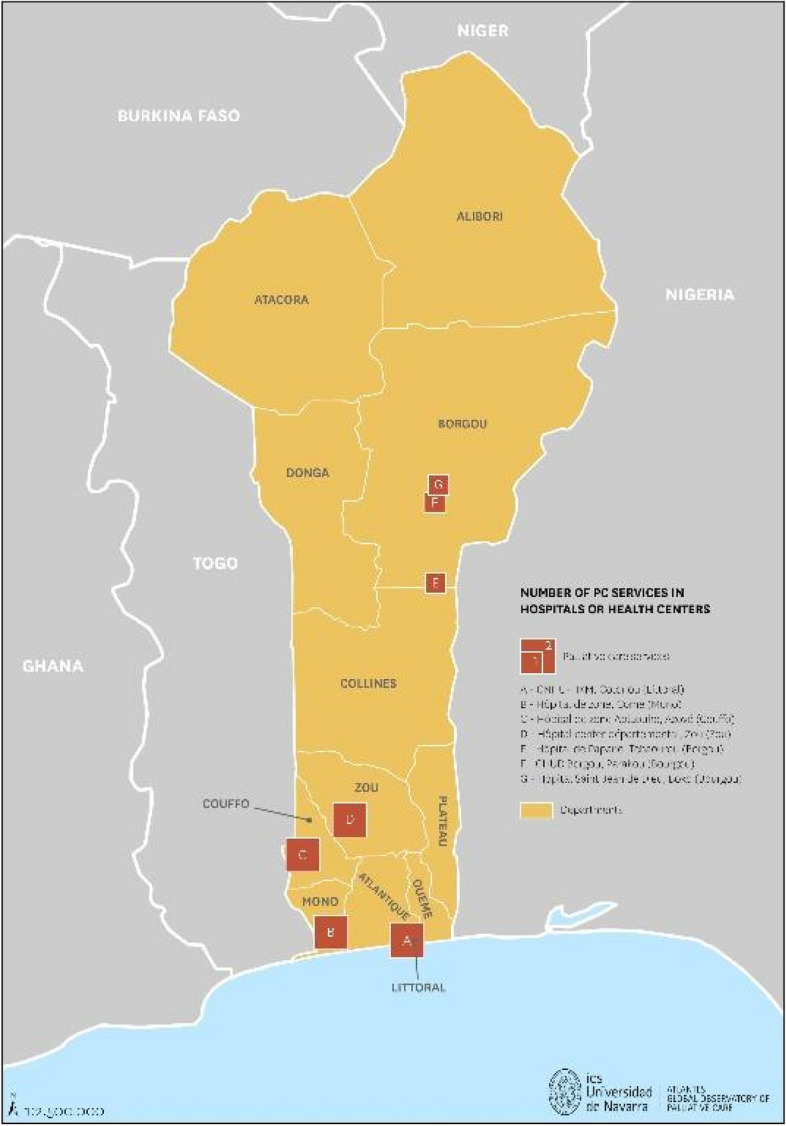
National distribution of palliative care teams

The evaluation in Benin reported eleven specialized palliative care teams are distributed as follows: Cotonou [2], Abomey [2], Aplahoué [2], Comé [2], Parakou [1], Boko [1], Papane [1] (Fig. 3). Most were hospital teams, but four teams provided care at home. According to the latest population data published by the World Bank (12,996,895), the ratio of services is 0.08 per 100,000 inhabitants. The teams included at least one doctor, one nurse, and one driver, and sometimes, they also included volunteers and students. Although there are no specialized pediatric palliative care teams, Benin has two pediatric oncologists trained with the help of GFAOP (Groupe Franco-Africain d’Oncologie Pédiatrique) and a pediatric oncology unit located in the Centre Hospitalier Universitaire Départemental-Ouémé-Plateau in the city of Porto-Novo [8]. According to the Beninese Society of Pediatrics (SoBePed), at their 5th Congress of to be held in Cotonou from 23 to 26 May 2023, a joint session between the Pediatric Oncology Unit of CHUD-OP, GFAOP and SoBePed to promote pediatric palliative care is planned.

## Discussion

The results of this evaluation revealed an opportunity for political and social will in Benin for the development of palliative care. Political commitment seems strongly settled thanks to the creation of the national five-year palliative care program 2022–2026 [8], which aims to work in four principal directions: axis (1) strengthening governance, leadership, partnership, and mobilization of financial resources; axis (2) improving the availability of quality human resources in palliative care at all levels of care; axis (3) development of the supply of quality palliative care at all levels of the health system, including in the community and at home; and axis (4) the availability of health products, technology, infrastructure, and other equipment for the provision of care at all levels. These four strategic axes match the main challenges reported in this evaluation: the scarce provision and coverage of both specialized and generalist services, the nearly non-existent PC education, and the unavailability and inaccessibility to the essential medicines for palliative care (even if oral morphine solution is produced and given to patients for free in some hospitals). Likewise, social will appears to be catalyzed by the many associations devoted to defending and advocating patients ´rights to palliative care. As presumed in the latest conceptualization of palliative care development, social involvement and partnership in health is the basis for the foundation of palliative care [18].

While palliative care-oriented research has not yet advanced in Benin (except for some isolated clinical literature [11, 17]), the organization, in October 2022, of the first congress on palliative care in Cotonou by the ABSP and PNSP, shows interest in research and allows the dissemination of knowledge about PC. In fact, research in due course is shown in the abstract book and at least one is already an “in press” scientific article on the clinical socioeconomic profile of patients followed by specialized PC teams in Benin [16]. As a result of the workshop, several participants agreed to constitute a PC Research group from which this study could be considered the first result.

Compared to the 2017 evaluation [10], the number of teams increased from 4 to 11, reflecting little access to PC for citizens. Despite the better availability of opioid medicines across health levels, consumption timidly rises from 0.14 mg to 0.18 mg per capita, which is well below the already low African average (1 mg/capita/year) or global average (31.1 mg/capita/year). A noticeable change in political will, a greater partnership from society in PC, or these timid net improvements detected in 2017, are insufficient to balance great shortcomings in education, research, access to essential medicines, and training for future healthcare professionals.

African reality generally shows a similar situation, characterized by an uneven distribution of PC services across the continent (concentrated in Uganda, South Africa, Rwanda, and Kenya) [19–22], about 20% of countries lacking a single PC service, and very low opioid consumption [10]. One notable finding of published African literature is that there is no information on PC provision for about half of African countries, and where data are available, these pertain mostly to Anglophone African countries [20]. This point is reflected in work carried out by advocacy groups such as Human Rights Watch, that confirmed that Francophone countries were lagging far behind in development compared with Anglophone countries [23].

Indeed, in an ongoing review on PC progress over French-speaking countries, all show little provision of services (ranging from 0 to 7) and an estimated opioid consumption of less than 1 mg per capita. In this context, Benin seems to have not only a more favorable policy situation, but also a slightly major provision of services and, importantly, a unit producing morphine that only happens in a few French-speaking countries like Rwanda and Senegal [24]. Benin could, to some extent, become a good practice example for these countries in terms of the five-year PC plan, assigned budget, and the activity of PC champions (as reported by other studies in other countries [25]).

Ultimately, although this study suggests that WHO indicators of palliative care provide an easily available and replicable methodology (as per another study conducted in India) [26], caution should be exercised when interpreting this overall regarding comparability across countries. The fact that indicators have been edited and need country adaptions could be a source of disparity if these adaptions vary widely from one country to another. In the case of Benin, the adjustments made were minor (mostly language-related) and did not deviate from the initial meaning of the indicators, which would permit an easy comparison. Future research should test the validity and capacity of this set of indicators and, with this aim, the World Health Organization is still piloting the set of indicators with identical methods in Morocco and Uruguay.

## Conclusion

This assessment highlighted the strengths and weaknesses as well as areas for improvement in public policy. Despite growing political, professional, and community commitments, there are shortcomings in education, research, access to essential medicines, and training for future healthcare professionals. The level of availability of PC teams still reflects very poor access to PC for citizens.

### Supplementary Information

Supplementary Material 1: Supplementary Table 1. Participants in the meeting with Benin´s key stakeholders to explain, validate and adapt the indicators to country's needs. Supplementary Table 2. WHO palliative care Indicator’s rating. Supplementary Table 3. Agreed data sources for the WHO palliative care indicators in Benin.

## Data Availability

No datasets were generated or analysed during the current study.
