# Correction: Palliative care progress in Benin: a situation analysis using the WHO development indicators

**DOI:** 10.1186/s12904-024-01500-9

**Published:** 2024-07-09

**Authors:** Kouessi Anthelme Agbodande, Freddy Gnangnon, Mickael Assogba, Josué Avakoudjo, Angèle Azon Kouanou, Lisette Odoulamy, Jean Daho, Djimon Marcel Zannou, Sourakatou Salifou, Ali Imorou Bah Chabi, Raoul Saizonou, Issimouha Dille Mahamadou, Fernanda Bastos, Eduardo Garralda, Carlos Centeno, Vilma Adriana Tripodoro

**Affiliations:** 1 Programme National des soins Palliatifs (PNSP/MS), Cotonou, Bénin; 2 Service de médecine interne -Centre National Hospitalo-Universitaire CNHU-HKM, Cotonou, Benin; 3 Médecin Point focal Cancer FSS/UAC/PNLMNT/MS, Cotonou, Benin; 4 Société Béninoise de lutte contre le Cancer, Cotonou, Benin; 5 Unité de Soins Palliatifs -Centre National Hospitalo-Universitaire CNHU-HKM, Cotonou, Benin; 6 Faculté des Sciences de la Santé, Cotonou, Benin; 7 Association Béninoise de soins palliatifs (ABSP), Cotonou, Bénin; 8 Institut national Médico-Sanitaire (INMeS), Cotonou, Benin; 9 Directeur départemental de la santé du Couffo, Aplahoué, Benin; 10 Conseil National de la Médecine Hospitalière (CNMH), Cotonou, Benin; 11 Directeur National de la Santé Publique (DNSP), Cotonou, Benin; 12 Secrétaire Général du Ministère SGM/MS, Cotonou, Benin; 13 National Professional Officier/NCD; OMS, Cotonou, Benin; 14 Médecin Technical officer Cancer for West and Central, Africa OMS/AFRO Unité MNT/AFRO Cluster (UCN), Dakar, Sénégal; 15 ATLANTES Global Observatory of Palliative Care, Institute Culture and Society, University of Navarra, Navarra, Spain

**Correction: BMC Palliat Care 23, 141 (2024)**

10.1186/s12904-024-01473-9.

Following publication of the original article [1], the authors requested to update the name of the author „Carlos Centeno Cortés“ into „Carlos Centeno”.

The author group has been updated above and the original article [1] has been corrected.
