# Supplementary material for: Palliative care progress in Benin: a situation analysis using the WHO development indicators
Source: BMC Palliat Care. 2024 Jun 5;23:141. doi: 10.1186/s12904-024-01473-9 (PMC11151610; doi:10.1186/s12904-024-01473-9)
Supplement: Supplementary file 1 — Supplementary Material 1: Supplementary Table 1. Participants in the meeting with Benin´s key stakeholders to explain, validate and adapt the indicators to country's needs. Supplementary Table 2. WHO palliative care Indicator’s rating. Supplementary Table 3. Agreed data sources for the WHO palliative care indicators in Benin. [file 12904_2024_1473_MOESM1_ESM.pdf]

Supplementary Table 1: Participants in the workshop

N°	Full name	Qualification and institution	Origin (town)	Profile	Feb 14	Feb 15
01	GNANGNON Freddy Houehanou Rodrigue	Associate Professor of Surgical Oncology , Cancer Focal Point School of health Sciences University of Abomey-Calavi National Program for the fight against NCDs SoBECAN: Beninese society of Oncology.	Cotonou	Consultant	√	√
02	BOKO KOULIDJI M. Reine Stéphanie	Administrative Assistant, in charge of Monitoring and Evaluation for the National Palliative Care Programme	Cotonou	Ministry of Health	√	√
03	SOGBOHOSSOU Paulin	Medical Doctor/ P@sris Enabel	Cotonou	Belgian technical cooperation	√	√
04	DAHO Jean Yaovi	Medical Doctor / Department Director of Health, Couffo	Couffo	Ministry of Health	√	√
05	ODOULAMI Lisette	Full Professor of ophthalmology; Director of the health national institute training school for nurses and midwives (INMeS)	Cotonou	University support	√	X
06	ZANNOU Djimon Marcel	Full Professor of internal medicine, President of the National Council for Hospital Medicine	Cotonou	Ministry of Health	√	√
07	SALIFOU Sourakatou	Medical Doctor, National Director of Public Health	Cotonou	Ministry of Health	√	X
08	IMOROU BAH CHABI Ali	Medical Doctor, Secretary General of the Ministry, Ministry of Health	Cotonou	Ministry of Health	√	X
09	BISSOUMA-LEDJOU Tania René	WHO representative	Cotonou	WHO	√	X
10	CENTENO Carlos	ATLANTES WHO Collaborating Centre for the Global Monitoring of Palliative Care Development	Spain	Technical office	√	√
11	GBENOUTIN BADE F. Jean de Dieu	Director of Information Systems, Ministry of Health	Cotonou	Ministry of Health	√	X

12	TOUME Cosme	Epidemiologist National Programme for Non-communicable diseases	Cotonou	Ministry of Health	√	√
13	AGUEGUE Aline	Pharmacist, Head of the morphine oral solution production unit, National Teaching Hospital(CNHU-HKM)	Cotonou	National Teaching Hospital(CNHU-HKM)	√	X
14	KOUANOU Angèle	Full Professor of internal medicine, Head of Internal Medicine Department, National Teaching Hospital (CNHU-HKM), and Beninese National Association for Palliative Medicine	Cotonou	National PC Association and clinician	√	√
15	DILLE MAHAMADOU Issimouha	Medical Doctor, Technical officer Cancer for West and Central Africa OMS/AFRO MNT/AFRO Unit Cluster (UCN)	Ouagadougou	WHO	√	√
16	SAIZONOU Raoul	Medical Doctor, National Professional Officer/NCD; WHO Benin	Cotonou	WHO	√	√
17	TRIPODORO Vilma	ATLANTES WHO Collaborating Centre for the Global Monitoring of Palliative Care Development	Pamplona	Technical office	√	√
18	GARRALDA Eduardo	ATLANTES WHO Collaborating Centre for the Global Monitoring of Palliative Care Development	Pamplona	Technical office	√	√
19	AGBODANDE K. Anthelme	Associate Professor of internal medicine, National Coordinator of the National Palliative Care Programme, Ministry of Health	Cotonou	Ministry of Health	√	√
20	AVAKOUDJO Josue	Full Professor of Urology, Dean of the Cotonou School of Health Sciences, University of Abomey Calavi	Cotonou	University support	√	X
21	OKE C. Severin	Information Systems, Ministry of Health	Cotonou	Ministry of Health	√	√
22	SOUSSIA Théodore	PhD/Masters Coordinator, health	Cotonou	University support	√	X

		national institute training school for nurses and midwives				
23	BOUESSEAU, Marie-Charlotte	Integrated health Services, HQ Geneva, WHO	Geneva	WHO	√	X
24	MONTERO Álvaro	ATLANTES WHO Collaborating Centre for the Global Monitoring of Palliative Care Development	Pamplona	Technical office	√	X
25	BASTOS Fernanda	ATLANTES WHO Collaborating Centre for the Global Monitoring of Palliative Care Development	Pamplona	Technical office	√	X
26	BASILIDA Romuald	Student		/	√	X
27	GOUNFLÉ Darius	Director departmental Hospital of Zou,	Abomey	Clinician	√	X
28	HOUANSOU Telesphore	WHO Benin	Cotonou	WHO	√	X
29	ZON	Student		WHO	√	X
30	ASSOGBA Mickael	Head of Palliative Care Unit - National University Hospital C (CNHU) Oncopediatrics CNHU-HKM Cotonou	Cotonou	Clinician	√	X

Supplementary Table 2: WHO palliative care Indicator's rating

	INDICATORS	Criterion	Median	Content validity index	Index of disagreement IPRAS)
EMPOWERMENT OF PEOPLE AND COMMUNITIES	1. Existence of groups promoting the rights of patients in need of palliative care, families, carers and survivors of illness	Relevance	5,0	0,85	0,24
		Feasibility	4,0	0,23	0,58
	2. Existence of a national policy or guideline on advance planning for medical decisions regarding the use of life-sustaining therapies or end-of-life care	Relevance	5,0	0,85	0,09
		Feasibility	5,0	0,54	0,24
HEALTH POLICIES	3. Existence of a national strategic plan for palliative care with a well-defined implementation framework	Relevance	5,0	1,00	0,00
		Feasibility	5,0	1,00	0,00
	4. Inclusion of palliative care in the list of health services provided at the primary care level in a package of priority services for universal health coverage in the national health system	Relevance	5,0	1,00	0,00
		Feasibility	4,0	0,08	0,37
	5. Existence of a national coordinating authority for palliative care (unit, service or department) within the Ministry of Health or an equivalent body responsible for palliative care	Relevance	5,0	1,00	0,00
		Feasibility	5,0	1,00	0,00
RESEARCH	6. Existence of national congresses or scientific meetings specifically dedicated to palliative care	Relevance	5,0	0,85	0,24
		Feasibility	5,0	0,69	0,24
	7. Research on palliative care relevant to the country: estimate based on the number of peer-reviewed articles	Relevance	4,0	0,69	0,24
		Feasibility	3,0	-0,54	0,00
	7.1. Existence of a research group dedicated to palliative care and which is officially recognized in the country	Relevance	5,0	0,85	0,24
		Feasibility	4,0	0,54	0,24
MEDICINES	8. Reported annual consumption of opioids - excluding methadone - in oral morphine equivalent (OME) per person	Relevance	5,0	1,00	0,09
		Feasibility	4,0	0,69	0,24
	9. Availability of essential pain and palliative care medicines in the country at all levels of care (estimate)	Relevance	5,0	0,85	0,00
		Feasibility	4,0	0,08	0,58
	10. Overall availability of immediate-release oral morphine (liquid or tablet) at primary care level (estimate)	Relevance	5,0	0,85	0,00
		Feasibility	3,0	-0,23	0,58
EDUCATION	11. Proportion of medical and nursing schools integrating palliative care training into their core curriculum	Relevance	5,0	0,85	0,00
		Feasibility	4,0	0,54	0,11
	12. Specialisation in palliative medicine for physicians	Relevance	4,0	0,23	0,58
		Feasibility	4,0	0,08	0,59
SERVICES	13. Number of specialised PC teams (nationwide) in relation to population	Relevance	5,0	0,85	0,09
		Feasibility	4,0	0,23	0,58
	14. Number of specialised paediatric PC teams in the country in relation to the population	Relevance	5,0	0,54	0,09
		Feasibility	3,0	-0,08	0,47

Median (1-5)	
≥80%	4 to 5
70%-79%	3,5 to 4
≤69%	0 to 3,4
**Content validity index (0-1)**	
≥0,70	≥0,70
0,31-0,69	0,31-0,69
≤0,30	≤0,30
**Disagreement index (0-1)**	
≤0,30	≤0,30
0,31-0,60	0,31-0,60
≥0,60	≥0,60

Supplementary Table 3: Agreed data sources for the WHO palliative care indicators in Benin

Indicators	Data sources	Data collection
1. Existence of groups promoting the rights of patients in need of palliative care, families, carers and survivors of illness	Registry of the National PC Program and the National NCDs Program (both within the MOH)	Consultation by the consultant
2. Existence of a national policy or guideline on advance planning for medical decisions regarding the use of life-sustaining therapies or end-of-life care	Legal adviser of the Ministry of Health (program, law, other policies).	In Benin, law 2020 -37, February 3rd 2021, on health protection
3. Existence of a national strategic plan for palliative care with a well-defined implementation framework	National 5 year palliative care plan: 2022-2026	Consultation by the consultant and ATLANTES
4. Inclusion of palliative care in the list of health services provided at the primary care level in a package of priority services for universal health coverage in the national health system	National Community Health Policy	Consultation by the consultant and ATLANTES
5. Existence of a national coordinating authority for palliative care (unit, service or department) within the Ministry of Health or an equivalent body responsible for palliative care	Ministry of Health	Consultation with Dr. Anthelme Agbodande (Coordinator of the National PC programme under MoH)
6. Existence of national congresses or scientific meetings specifically dedicated to palliative care	Report of the first National palliative Care congress in Benin	Consultation with Dr. Anthelme Agbodande (Coordinator of the National PC programme under MoH, and Prof Angele Kouanou (President of the National PC Association)
7. Research on palliative care relevant to the country: estimate based on the number of peer-reviewed articles	Biomedical databases (Pubmed, Cinahl, Embase). Additionally grey literature in google.	Background scoping review
8. Existence of a research group dedicated to palliative care and which is officially recognized in the country	National Palliative Care Program<br><br>Both universities in the country (University of Abomey Calavi and University of Parakou)	Consultation by the consultant
9. Reported annual consumption of opioids - excluding methadone - in oral morphine equivalent (OME) per person	International Narcotics Control Board (INCB). Governments report the amount of pursued opioids in a given year to the INCB.	Data are published by the Walther Global Palliative Care Center (Indiana University). There is a country report for Benin.
10. Availability of essential pain and palliative care medicines in the country at all levels of care (estimate)	*Ad hoc* survey with the list of essential medications of the WHO available.	Survey to all 34 coordinating doctors of the diverse health areas of Benin by the consultant

11. Overall availability of immediate-release oral morphine (liquid or tablet) at primary care level (estimate)	National Palliative Care Program	Consultation by the consultant
12. Proportion of medical and nursing schools integrating palliative care training into their core curriculum	Direct contact with the country's medical and paramedical schools (and university websites)	Survey to all universities and nursing schools by email and phone by the consultant
13. Specialization in palliative medicine for physicians	Ministry of Health and Ministry of Higher Education	Survey to all universities and MOH
14. Number of specialized palliative care teams (nationwide) in relation to population	*Ad hoc* Consultant (after compliance between the Ministry of Health and the National Palliative Care Association)	Consultation by the consultant
15. Number of specialized pediatric palliative care teams in the country in relation to the population	*Ad hoc* Consultant (after compliance between the Ministry of Health and the National Palliative Care Association)<br><br>Beninese Society of pediatrics (SoBePed)	Consultation by the consultant

Supplementary table 4

Questionnaire for the country survey

Indicator 1. Existence of groups responsible for promoting the rights of patients in need of palliative care, families, carers and survivors of illnesses

Please tick the box (□) most appropriate for your country.

**Component**	**Level 1**<br><br>**Early stage**	**Level 2**<br><br>**Intermediate stage**	**Level 3**<br><br>**Established stage**	**Level 4**<br><br>**Advanced stage**
**Existence of groups responsible for promoting the rights of patients in need of palliative care, families, carers and survivors of illnesses**	□ Only isolated activity was observed	□ Precursors, promoters or defenders of palliative care can be	□ Existence of one or more groups that cover palliative care in a more integrated way or over a greater number of	□ A strong national and sub-national presence to defend palliative

		identified, but no formal organisation has been set up.	diseases/programme areas	care and promote patients' rights (for example, in the form of a professional palliative care association).

Definitions

Associations or groups responsible for representing patients receiving palliative care and their carers and for defending and promoting their rights. They provide legal representation, participation, defence and consultation for users and advise them in their field of action. Associations of family members of children receiving palliative care are also involved.

Main data sources

Key informant surveys with stakeholders from the Department of Health and/or the National Hospice and Palliative Care

References

- Pettus, K. I., & de Lima, L. (2020). Palliative Care Advocacy: Why Does It Matter? *Journal of Palliative Medicine*, *23*(8), 1009-1012.

- Clark, J., Barnes, A., & Gardiner, C. (2018). Reframing global palliative care advocacy for the sustainable development goal era: a qualitative study of the views of international palliative care experts. Journal of Pain and Symptom Management, 56(3), 363-

Indicator 2. Existence of a national policy or guideline on advance planning for medical decisions regarding the use of life-sustaining therapies or end-of-life care

Please tick the box (□) most appropriate for your country.

**Component**	**Level 1**<br><br>**Early stage**	**Level 2**<br><br>**Intermediate stage**	**Level 3**<br><br>**Established stage**	**Level 4**<br><br>**Advanced stage**
**Is there a national policy or guideline on advance care planning or advance directives? (Choose the highest level that applies).**	□ Lack of national policy or guidelines on advance care planning	□ Existence of one or more national policies or guidelines concerning **substitute decision-makers**	□ Existence of one or more national policies or guidelines concerning **living wills** and/or<br><br>advance directives	□ Existence of a national policy on **advance care planning**

Definitions

In order to participate actively in decision-making, people facing serious or life-threatening illnesses should have the opportunity, at an early stage: (i) to receive transparent information about life-sustaining therapies and palliative care; and (ii) to express in advance their values and preferences regarding the life-sustaining therapies and care they will receive. The advance care planning process should also involve family members.

- A **subrogated decision-maker**, also known as a health care proxy or representative, is a **person** appointed to represent patients who do not have the capacity to do so. If a patient does not have the capacity to make decisions about their own personal care, a substitute decision-maker must make the decisions for them.
- A **living will**, also known as an **advance directive**, is a legal **document** that specifies the type of medical care a person wishes or does not wish to receive in the event that they are unable to communicate their wishes.
- **Advance care planning** is the **process of** planning for future health care. It concerns the health care that a person would or would not wish to receive if they were to become seriously ill or injured and unable to communicate their preferences or make decisions. It usually concerns the care a person will receive at the end of life. Advance care planning allows people to think about, discuss and record their preferences regarding the type of care they wish to receive and the consequences they consider acceptable. Ideally, advance care planning will result in the person's preferences being recorded in a plan known as **advance directives** and in the appointment of a **proxy** to ensure that their preferences are respected.

Main data sources

Official source at the Ministry of Health

References

- Rietjens, J. A., Sudore, R. L., Connolly, M., van Delden, J. J., Drickamer, M. A., Droger, M., ... & European Association for Palliative Care. (2017). Definition and recommendations for advance care planning: an international consensus supported by the European Association for Palliative Care. *The Lancet Oncology*, *18*(9), e543-e551.

Indicator 3. Existence at national level of a palliative care plan, programme, policy or strategy in force with a well-defined implementation framework.

Please tick the box (□) most appropriate for your country.

Criteria	Level 1<br>Early stage	Level 2<br>Intermediate stage	Level 3<br>Established stage	Level 4<br>Advanced stage
**1. Existence of a national palliative care plan, programme, policy or strategy**<br><br>***Note:*** *This national plan/programme/policy/strategy may be part of the national strategy or a stand-alone strategy. If there is no national palliative care plan, programme, policy or strategy, please select "None" and go to the next indicator.*	□ Absence	□ Developed over 5 years ago	□ Updated in the last 5 years, but not actively assessed or audited	□ Updated within the last 5 years and actively assessed or audited
**2. Existence of a validated national strategic orientation on palliative care including the following criteria:**<br><br>**Note**: *the average of the individual component scores below is calculated to obtain the overall score for the criterion.*	No criteria met, or only minimum criteria met	Minimum orientation towards palliative care	Limited focus on palliative care	The national strategy for palliative care is fully focused
a. The national palliative care plan (or programme,	□ Don't know	□ Absence of a national palliative	□No, but another national	□Yes, existence of an independent national palliative care plan

strategy or legislation) is independent.		care plan. whether independent or integrated into another national plan	plan, such as a national plan to combat cancer, NCDs or HIV, contains a section specifically devoted to palliative care.	AND/OR legislation/government decrees on palliative care
b. L'validated national strategic guideline on palliative care has a guidance with a well-defined set of quality palliative care interventions *(1)*	☐ Does not have defined high-quality palliative care interventions	☐ Has a well-defined intervention in quality palliative care	☐ Has a well-defined intervention in quality palliative care	☐ Has a comprehensive range of interventions focused on quality palliative care
c. Guidance resulting from a consultation process involving communities	☐ Little or no stakeholder involvement	☐ Emerging stakeholder involvement	☐ Broad involvement (but absence of certain key groups)	☐ A full consultative process has been carried out, also involving the communities.

**Criteria**	**Level 1**<br>**Early stage**	**Level 2**<br>**Intermediate stage**	**Level 3**<br>**Established stage**	**Level 4**<br>**Advanced stage**
d. Publication and distribution of the final version (independently or as part of a national health strategy)	☐ Version in preparation	☐ Draft version	☐ Final version published, but not yet released	☐ Final version published and distributed

e.	Direction recognised by senior health officials as it has been finalised and validated (the necessary governance stages to be validated for implementation having been completed)	☐ Orientation in progress, but not recognised by senior management as being finalised	☐ Orientation completed, but not recognised by senior management as being finalised	☐ Guidance recognised by senior health officials as finalised, but not validated for implementation	☐ Guidance recognised by senior health officials as finalised and validated for implementation
f.	Directorate, department, unit or recognised focal point for palliative care responsible for developing and implementing national guidance on palliative care	☐ Absence	☐ There are recognised palliative care units, but they are relatively few in number	☐ Palliative care units are recognised and operational, but need to be strengthened	☐ Existence of a fully operational palliative care unit capable of implementing national palliative care plans
g.	Existence of mechanisms for monitoring and evaluating progress, with measurable objectives.	☐ Absence	Existence of mechanisms for monitoring and evaluating progress, with clear objectives, but they have not yet been implemented	Mechanisms exist but have not been updated (implemented outside the specified period)	Existence of mechanisms for monitoring and evaluating progress, with measurable objectives, currently being implemented
h.	Clear description of plans, processes or systems for reaching and meeting the needs of key affected and at-risk populations	☐ No mention in the national plan or strategy	Only one **mention** to cover the needs of all key affected populations (i.e. cancer and non-cancer patients)	Explicit **reference to** coverage of the needs of the main **populations** affected (cancer and others) **and at** risk	Detailed description of plans, processes and systems for reaching and meeting the needs of the main populations affected and at risk

(1) According to the technical document published by the WHO, interventions focusing on quality of care would be integrated into palliative care efforts

Definitions

The national plan or programme or strategy refers to regulatory and official publications that are applicable throughout the country (these may be laws or other official government documents). These publications are usually approved by the national health authority.

This indicator refers to any official government document that includes information on palliative care. The following three non-exclusive options are possible:

- The **national law on palliative care or any other specific legislation or government decree relating to certain features of palliative care** includes, but is not limited to, the regulation of service provision, organisation, accessibility, information, transport, dependency, family allowances, etc. In some legislations, reference is made to palliative care as a human right, or in others, the inclusion of teaching palliative care to medical students is required by law.
- An **independent national programme on palliative care** is defined as a specific plan or programme distinct from other national health plans. It usually contains norms and criteria for the development of palliative care, regulations for service provision and, in some cases, guidelines for palliative care research. A national plan/programme must be national in scope, designed to integrate palliative care into health care services and have a budget and a responsible person.

Main data sources

- Official source at the Ministry of Health

- Key informants

References

- Clelland, D., van Steijn, D., Whitelaw, S., Connor, S., Centeno, C., & Clark, D. (2020). Palliative care in public policy: results

Indicator 4. Inclusion of palliative care on the list of health services provided at primary care level in a package of priority services for the purposes of universal health coverage in the national health system

Please tick the box (□) most appropriate for your country.

Criteria	Level 1<br>Early stage	Level 2<br>Intermediate stage	Level 3<br>Established stage	Level 4<br>Advanced stage
**Palliative care services are included on the list of health services provided at primary care level for the purposes of universal health coverage in the national health system.**	□ Under no circumstances	□ Existence of a decree or law on the inclusion of palliative care on the list of health services provided at primary care level in preparation	□ Palliative care included on the list of essential services recognised by a government decree or law, but not in the General Health Act.	□ Palliative care included on the list of health services provided at primary care level in the General Health Act
**Access to these services is monitored regularly and is broken down by gender and socio-economic status.**	□ No mechanism for monitoring access to these services	□ Existence of a mechanism for monitoring access to these services, but it is not operational	□ Regularly monitored access to these services	□ Access to these services regularly monitored and broken down by gender and socio-economic status

Definitions National health systems design, approve and implement a package of basic health services for universal health coverage. The aim of this package is to achieve MDG 3.8, the target of which is to ensure that everyone has universal health coverage, including financial risk protection and access to quality essential health services and to safe, effective, quality and affordable essential medicines and vaccines. For this study, palliative care services should be explicitly mentioned at the primary health care level in the package of priority services for universal health coverage.

The inclusion of palliative care on the list of health services provided at primary care level is generally regulated by national health laws or other government decrees. Countries draw up a catalogue of services stipulating the services that should be available and provided at primary care level in their territory. One of the services to be included on the list should be palliative care.

This indicator only assesses the inclusion of palliative care on the list of services provided in primary care, but not their implementation. To answer "yes" to this indicator, and tick the box corresponding to level 2, 3 or 4 of this indicator, the specific term palliative care must appear on this list.

Indicator 5. Existence of a national palliative care coordinating authority (unit, service or department) within the Ministry of Health or an equivalent body responsible for palliative care.

Please tick the box (☐) most appropriate for your country.

Criteria	Level 1<br><br>Early stage	Level 2<br><br>Intermediate stage	Level 3<br><br>Established stage	Level 4<br><br>Advanced stage
**1. Is there a national palliative care authority within the government or the Ministry of Health?**<br><br>**Note**: If there is *no identifiable coordinating body for palliative care, please select "No coordinating body" and move on to the next indicator.*	☐ No coordinating body	☐ Authority for palliative care defined, but only at political level, with no defined coordinating body	☐ Coordination entity with an incomplete structure (no scientific or technical section)	☐ Well-defined palliative care coordination entity with a good structure (scientific and technical)
**2. The national coordinating authority or authorities are responsible for coordinating, monitoring, integrating and implementing national palliative care strategies and policies.**	☐ Lack of national authority/coordination function	☐ Palliative care mentioned in political speeches by the Ministry of Health	☐ National coordinating authority in operation (but in need of strengthening)	☐ National coordinating authority operating and actively implementing a work programme
**3. The national coordinating authority or authorities shall have the appropriate authority, budget and staff.**	☐ Lack of appropriate authority or resources (budget, staff, etc.)	☐ No budget, and staff or the authority are faced with major shortfalls	No budget, but sufficient staff and authority	☐ Existence of an appropriate authority, budget and staff
**4. Existence of sub-national/sub-regional authorities with operational capacity**	☐ Lack of sub-national/sub-regional operational capacity	☐ Existence of sub-national/sub-regional operational capacity in some	☐ Existence of sub-national/sub-regional operational capacity in almost all regions, but some have	☐ Existence of a strong sub-national/sub-regional operational capacity in all

		regions, but not in most	weak operational capacity	regions and in all areas
**5. Mechanisms for engaging vulnerable groups with the authorities are defined**	☐ No defined mechanism	☐ Mechanism defined but not implemented	☐ Mechanisms have been put in place, but have only been put into practice belatedly	☐ Existence of defined, implemented and currently operational mechanisms

Definitions

The national authority responsible for palliative care policy may be organised in different ways in different countries. The basic principle is that one person in the political structure of the ministry has the highest assigned responsibility **(political authority**: a director or deputy director general, for example). In addition, in some countries, one or more health technicians on the ministry's staff are responsible for the **technical tasks** of managing or evaluating palliative care. Similarly, it is common for the coordination department to appoint one or more palliative care professionals to carry out **scientific advisory tasks**.

Breakdown according to the role(s) played by the national authority(ies) concerning palliative care services and activities, i.e. :

- coordination, - monitoring and evaluation - implementation of national policy/strategy - budget holders - provision

Main data sources

Official source at the Ministry of Health.

References

Palliative Care integration in national health systems in Europe. In Arias-Casais, N., Garralda, E., Rhee, J. Y., Lima, L., Pons-Izquierdo, J. J., Clark, D., ... & Centeno, C. (2019). EAPC Atlas of Palliative Care in Europe 2019.

Indicator 6. Existence of national congresses or scientific meetings specifically devoted to palliative care

Please tick the box (☐) most appropriate for your country.

**Component**	**Level 1**<br><br>**Early stage**	**Level 2**<br><br>**Intermediate stage**	**Level 3**<br><br>**Established stage**	**Level 4**<br><br>**Advanced stage**
**Existence of scientific meetings or congresses specifically devoted to palliative care <u>in the</u>** country	☐ Absence of national conferences or scientific meetings related to palliative care	☐ Palliative care conferences or meetings are held only sporadically (irregularly).	☐ At least one congress or conference not directly devoted to palliative care (e.g. on cancer, HIV, chronic diseases, etc.), part of which is regularly devoted to palliative care, is held every 1 to 2 years (and no national conference specifically devoted to palliative care).	☐ At least one national conference specifically devoted to palliative care is held every three years

Definitions

The progress of research in a country can be illustrated by the hosting of different research activities, such as national congresses or scientific meetings (on the country's territory).

Main data sources

Key informant survey with National Hospice Palliative Care Association stakeholders (and any programme documentation, conference report).

Indicator nº7. Research on palliative care relevant to the country: estimate based on the number of articles submitted for peer review

Please tick the box (□) most appropriate for your country.

Question	Answer
**Number of peer-reviewed articles published in any language in the last 5 years with at least one author from the country of origin**	□□□ Number of items<br>□ Unknown

Definitions

They report results obtained using quantitative, qualitative or mixed methods of research into the prevalence, incidence, symptoms or relief of pain, including palliative care and pain (as terms). Similarly, the prevention or relief of physical, psychological, social or spiritual suffering associated with serious illness, or the systematic review of such studies with the keyword or search term "palliative care" or "hospice" in PubMed, CINHAL and Embase.

Main data sources

Scientific databases; PubMed; CINHAL, Embase

References

- Rhee, J. Y., Garralda, E., Torrado, C., Blanco, S., Ayala, I., Namisango, E., ... & Centeno, C. (2017). Publications on palliative care development can be used as an indicator of palliative care development in Africa. *Journal of Palliative Medicine*, *20*(12),

Indicator nº8. Reported annual consumption of opioids - excluding methadone - in oral morphine equivalent (OME) per person

Question	Answer	
**Notified annual consumption of opioids - excluding methadone - in oral morphine equivalent (OME) per person**	☐☐	Opioid consumption in milligrams per person per year, in oral morphine equivalent
	☐	Unknown

Definitions

Annual opioid consumption represents the quantity of opioids legally distributed for medical purposes in a country to institutions and programmes authorised to administer them to patients, such as hospitals, nursing homes, pharmacies, hospices and palliative care programmes.

Main data sources

Data on opioid consumption come from the most recent consumption figures reported to the International Narcotics Control Board (INCB)

References

Ju, C., Wei, L., Man, K. K., Wang, Z., Ma, T. T., Chan, A. Y., ... & Lau, W. C. (2022). Global, regional, and national trends in opioid analgesic consumption from 2015 to 2019: a longitudinal study. *The Lancet Public Health*, *7*(4), e335-e346.

Jayawardana, S., Forman, R., Johnston-Webber, C., Campbell, A., Berterame, S., de Joncheere, C., ... & Mossialos, E. (2021). Global consumption of prescription opioid analgesics between 2009-2019: a country-level observational study. *EClinicalMedicine*, *42*, 101198.

Indicators 9 and 10. Availability of essential pain and palliative care medicines in the country and general availability of immediate-release oral morphine (in liquid or tablet form) in primary care

★

Question	Answer
Percentage of health facilities at primary care level with pain and palliative care medicines as defined in the WHO Model List of Essential Medicines.	[ ][ ][ ] % of establishments with drugs available<br>[ ][ ][ ] Same % in urban areas<br>[ ][ ][ ] Same % in rural areas<br>[ ] Unknown
Percentage of primary care facilities with immediate-release oral morphine (in liquid or tablet form)	[ ][ ][ ] of establishments with medicines<br>[ ][ ][ ] Same % in urban areas<br>[ ][ ][ ] Same % in rural areas<br>[ ] Unknown

Definitions

Pain and palliative care medicines include:

- availability of non-opioids and non-steroidal anti-inflammatory drugs (NSAIDs) on the WHO Model List of Essential Medicines for Pain and Palliative Care (for children and adults)

- availability of other strong opioids on the WHO Model List of Essential Medicines for Pain and Palliative Care (for children and adults)

- availability of painkillers and palliative care medicines on the WHO Model List of Essential Medicines (for children and adults) used in palliative care to treat other symptoms

Main data sources

Facility surveys, such as Service Availability and Operational Capacity Assessment (SARA), Service Delivery Assessment (SPA), Service Delivery Indicator (SDI) surveys, Harmonised Health Facility Assessment (HHFA) and other stand-alone country assessments and/or Logistics Management Information Systems (LMIS).

Other data sources: Ministry of Health or country expert in its national healthcare package

References

- WHO model list of essential medicines, 21st list, 2019. World Health Organization; Geneva: 2019
- De Lima, L., Krakauer, E. L., Lorenz, K., Praill, D., Macdonald, N., & Doyle, D. (2007). Ensuring palliative medicine availability: the development of the IAHPC list of essential medicines for palliative care. Journal of pain and symptom

Indicator 11. Proportion of medical and nursing schools integrating palliative care training into non-graduate curricula

Question	Answer
**Total number of medical schools** in the country	Total number of medical schools<br>Unknown
Number of **medical schools** offering **compulsory courses** in palliative care (with or without other optional courses)	Number of medical schools offering compulsory courses in palliative care<br>Unknown
Number of **medical schools** offering **optional courses** in palliative care (without compulsory courses)	Number of medical schools offering optional courses in palliative care<br>Unknown
**Total number of nursing schools** in the country	Total number of medical schools<br>Unknown
Number of **nursing schools** offering **compulsory courses** in palliative care	Number of medical schools offering compulsory courses in palliative care

(with or without other optional courses)	☐ Unknown
Number of **nursing** schools offering **optional courses** in palliative care (without compulsory courses)	☐☐☐ Number of medical schools offering **optional** courses in palliative care<br>☐ Unknown

Definitions

Palliative care is taught to future doctors and nurses (as a compulsory or optional subject). A significant number of hours are devoted to this training.

Main data sources

- Ministry of Higher Education
- Official source at the Ministry of Health
- Key informant (survey)

References

- Carrasco, J. M., Lynch, T. J., Garralda, E., Woitha, K., Elsner, F., Filbet, M., ... & Centeno, C. (2015). Palliative care medical education in European universities: a descriptive study and numerical scoring system proposal for assessing educational development. *Journal of pain and symptom management*, *50*(4), 516-523.
- Noguera A, Bolognesi D, Garralda E, Beccaro M, Kotlinska-Lemieszek A, Furst CJ, Ellershaw J, Elsner F, Csikos A, Filbet M, Biasco G, Centeno C. How Do Experienced Professors Teach Palliative Medicine in European Universities? A Cross-Case Analysis of Eight Undergraduate Educational Programs. J Palliat Med. 2018 Nov;21(11):1621-1626.

Indicator no. 12. Specialisation in palliative medicine for doctors

Please tick the box (☐) most appropriate for your country.

**Component**	**Level 1** **Early stage**	**Level 2** **Intermediate stage**	**Level 3** **Established stage**	**Level 4** **Advanced stage**
**Existence of an official specialisation in palliative medicine for doctors, recognised by the competent national authority**.	☐ No official specialisation in palliative medicine for doctors	☐ No official specialisation in palliative medicine for doctors, but existence of other types of professional training diplomas **without official, national recognition** (i.e. advanced training courses or masters degrees in certain universities or institutions)	☐ No official specialisation in palliative medicine for doctors, but existence of other types of diploma with **official recognition** (for example, certification of the professional category or position of palliative care doctor).	☐ Existence of an official specialisation in palliative medicine, recognised by the competent national authority as a [illegible] **speciality, sub-speciality** [illegible] (or equivalent [illegible] designation)

Definitions

Specialisation programmes in palliative medicine here refer to all the conditions required to obtain the highest level of professional training in palliative medicine and official certification valid throughout the country.

Any specialty, sub-specialty or other term indicating formal certification for full-time palliative care physicians has been

Main data sources

Documents on national medical curriculum standards held by the Ministry of Health, the Ministry of Higher Education, the College of Physicians or equivalent, or others. Other sources of data: key informant survey

References

- Centeno, C., Bolognesi, D., & Biasco, G. (2015). Comparative analysis of specialization in palliative medicine processes within the World Health Organization European region. Journal of pain and symptom management, 49(5), 861-870.

Indicators 13 and 14. Number of specialised palliative care programmes (nationwide) and specialised paediatric palliative care programmes in the country in relation to the population.

Question	Answer
Please indicate the number of specialist palliative care programmes in the country	☐☐ Total number of specialist palliative care programmes in the country<br>☐ Unknown
Please indicate the number of specialised paediatric palliative care programmes in the country	☐☐ Total number of specialist palliative care programmes in the country<br>☐ Unknown

Please tick the box (□) most appropriate to your country's context. ***(Additional information in indicators 13 and 14*****).**

**Criteria**		**Level 1**<br><br>**Early stage**	**Level 2**<br><br>**Intermediate stage**	**Level 3**<br><br>**Established stage**	**Level 4**<br><br>**Advanced stage**
**1. Existence of a system of specialised palliative care programmes, with a geographical scope and provided by different service delivery platforms**<br><br>**Note**: *the average of the individual component scores below is calculated to obtain the overall score for the criterion.*		Absence or virtual non-existence of specialised palliative care programmes in the country	Isolated benefit: available, but only in certain geographical areas	Generalised benefit: available in many parts of the country, but with some gaps	Integrated service: specialist palliative care programmes offered as a matter of course
a. Existence of specialised paediatric palliative care programmes in the country		□ Under no circumstances	□ Occasional/in certain regions of the country	□ Present in many parts of the country	□ Strong presence in all regions of the country
b. Available to people living in urban areas		□ Under no circumstances	□ Occasional/in certain urban areas of the country	□ In many urban environments	□ In all urban environments
c. Available to people living in rural areas		□ Under no circumstances	□ Occasional/in certain rural areas of the country	□ In many rural areas	□ In all rural areas
d. Available in **public sector hospitals, for** example for hospital palliative care teams (responsible for consultations), palliative care units (with a certain number of beds), to name but a few examples.	□ N/A	□ Under no circumstances	□ Occasional/in certain regions of the country	In a growing number of public sector hospitals	□ Are part of most/all hospitals in one form or another
e. Available in **private sector hospitals, for** example for palliative care teams in hospitals (responsible for consultations), palliative care units (with a	□ N/A	□ Under no circumstances	□ Occasional/in certain regions of the country	In a growing number of private sector hospitals	□ Are part of most/all hospitals in one form or another

certain number of beds), to name but a few examples.					
f. **Independent hospices** (including hospices with inpatient beds)	☐ N/A	☐ Under no circumstances	☐ Occasional/in certain regions of the country	☐ Present in many parts of the country	☐ Strong presence of independent hospices in all regions of the country
**g. Home care teams** (specialising in palliative care) are available in the community (or at primary care level), as independent services or linked to hospitals or hospices.	☐ N/A	☐ Under no circumstances	☐ Occasional/in certain regions of the country	☐ Present in many parts of the country	☐ Strong presence of homecare teams in all regions of the country

Definitions

Palliative care services: specialised palliative care (PC) programmes are healthcare programmes whose main activity is to provide palliative care. They often provide care for patients who are suffering greatly or whose needs are complex, and therefore call on the services of specially trained carers.

Specialist paediatric palliative care programmes are programmes whose main activity is to provide palliative care to children. They often provide care for children who are in a great deal of pain or whose needs are complex, and therefore require specially trained carers.

The team usually includes at least one doctor and one nurse trained in palliative care, although in some areas a specialist palliative care programme is sometimes delivered by a single nurse with specialist training. Palliative care may be provided in, but is not limited to, independent hospices, hospices attached to a public or private sector hospital, any other type of hospice or by home care teams, palliative care teams in hospitals (providing consultation), palliative care units (with a certain number of beds), units serving hospice patients, etc.

In the context of this "palliative care programme" concept, any subdivision of the same programme is considered to be a separate palliative care programme: for example, a hospice with both a home care team and an inpatient unit will be considered to be offering two palliative care programmes.

Main data sources

- national palliative care directory

- district or national (health facility) databases available where registration of health services is mandatory

- survey of key informants

References

White Paper on criteria and standards for palliative care in Europe: part 1: available at: https://www.researchgate.net/publication/279547069_White_paper_on_standards_and_norms_for_hospice_and_palliative_care_in_Europe_Part_1 [accessed 8 October 2018]).
